# Editorial: Targeted therapy in advanced thyroid cancer

**DOI:** 10.3389/fendo.2022.1022698

**Published:** 2022-09-02

**Authors:** Laura Boucai

**Affiliations:** Department of Medicine, Endocrinology Service, Memorial Sloan Kettering Cancer Center, New York, NY, United States

**Keywords:** thyroid cancer, targeted therapy, immunotherapy, peptide receptor nuclide therapy, anaplastic thyroid cancer, medullary thyroid cancer

The last decade has seen a transformation in the management of thyroid carcinoma. New research supporting the surveillance of indolent disease and the use of targeted systemic therapy when the genomics of the tumor are known have resulted in a new era of therapeutic possibilities. Clinicians have now the ability to more precisely diagnose indeterminate nodules and predict the behavior of confirmed thyroid cancers. Understanding the prognostic factors associated with disease recurrence, and the molecular events that disrupt the intracellular MAP-kinase and PI3-kinase pathways equips practitioners with an array of novel treatment tools. Most recently, the interaction between the tumor, its bone marrow derived microenvironment, and the immune response of the host has been a major focus of research interest that attempts to shed light on the pathogenesis and progression of the disease.

Recent years have seen breakthrough discoveries in our ability to redifferentiate radio-iodine refractory tumors and treat medullary and anaplastic thyroid cancer by targeting the specific molecular events that give rise to the tumor. Additionally, acquired resistance to these therapies has emerged in the form of tumor heterogeneity or additional mutations accumulated over time and major efforts are being undertaken to overcome it. There is an unmet medical need to advance the field of drug development and targeted therapies in thyroid carcinoma by continuing to study the intracellular events that disrupt the normal signaling of cells and promote unregulated growth as well as the study of the tumor and its microenvironment.

This Research Topic includes a wide range of topics that address the most frequent questions encountered in recurrent and advanced thyroid carcinoma. The first set of manuscripts address the question of how to best predict the appearance of central lymph node metastases. Central compartment and lateral neck lymph nodes have historically been the first station of thyroid cancer spread after which extension to distant sites outside of the neck can occur. Understanding the factors that contribute to the presence of central lymphadenopathy may enable practitioners to adjust their surveillance strategies and in turn prevent progression of tumors to distant metastatic sites. Tan et al. use the clinical and tumor characteristics of 642 papillary thyroid carcinomas in 340 patients to build multivariate models that predict the presence of central lymph node metastases. They conclude that age younger than 40 years, male sex, and ultrasonographic characteristics including: irregular borders, microcalcifications, tumor location in the isthmus and abnormal cervical lymph nodes predict the occurrence of large volume central lymph node metastases whereas the presence of chronic lymphocytic thyroiditis is associated with a decreased risk of central lymphadenopathy. In a separate study, Zhao et al. determine that male sex and age are strong predictors of metastatic central compartment lymph nodes in clinical T1b disease while male sex, young age, tumor size, contact with the capsule, and multifocality independently predict central lymphadenopathy in intrathyroidal tumors smaller than 1cm is size. The use of molecular genetics to complement the knowledge obtained by clinical characteristics has been exemplified by Wu et al. who demonstrate in their novel study that a glycolysis risk score (GRS) outperformed conventional clinicopathologic characteristics for prediction of recurrence-free survival. They use the transcriptomic profile of 838 patients from 6 public datasets to screen for genes related to glycolysis. The authors integrate the predictive power of four glycolysis-related genes: ADM, MKI67, CD44 and TYMS with clinicopathological features to optimize the model to predict recurrence. The above studies demonstrate that knowledge and use of specific patient and tumor characteristics are essential to tailor the surveillance of thyroid cancer disease.

When the disease has spread and become refractory to the effect of radioactive iodine, targeted and non-targeted redifferentiation techniques are available to halt progression. In these series, Du et al. demonstrate the effectiveness of apatinib, a tyrosine kinase inhibitor that selectively inhibits the vascular endothelial growth factor receptor-2, to control the progression of radio-iodine refractory thyroid carcinoma in 44 patients. They describe a promising overall response rate of 76% with a favorable safety profile.

The use of non-targeted tyrosine kinase inhibitors has been effective but limited due to systemic toxicities including hypertension, diarrhea and occasional life-threatening events. Gubbi et al. describe a novel approach of peptide receptor radionuclide therapy (PRRT) for the treatment of patients with somatostatin receptor (SSTR) positive radio-iodine refractory differentiated thyroid carcinomas and medullary thyroid carcinomas (MTC) that has the potential of becoming adopted with a favorable safety profile. Matrone et al. present the evidence of current selective and non-selective therapies for the treatment of sporadic advanced metastatic progressive MTC. They describe that radiometabolic treatment with Lutetium-177 may only benefit a subset of patients with high somatostatin receptor expression and high uptake on 68Ga-DOTA-SSA PET/CT. They highlight the revolutionizing role and effectiveness of second generation selective RET inhibitors and discuss the promising efforts currently underway to develop third generation RET inhibitors that would overcome the resistance to the former.

Huang et al. evaluate the efficacy and safety of Lenvatinib in a meta-analysis of 10 studies with patients harboring anaplastic thyroid carcinoma (ATC). They note that while Lenvatinib may have some anti-tumoral effect, it has limited efficacy in the treatment of ATC due to short, pooled progression-free and overall survival.

Finally, Garcia-Alvarez et al. present the current evidence on the effect of immunotherapy in thyroid cancer. They highlight the role of immunotherapy in ATC with an ORR in patients receiving immune checkpoint inhibitors ranging from 19% to 75%, with some patients achieving a complete response (CR). The authors note that efficacy of these agents in differentiated thyroid carcinoma has been more modest and further investigations are needed to define the specific subpopulations that may benefit from these agents.

All in all, this Research Topic underscores the progress made toward a personalized approach in the management of thyroid carcinoma with new and exciting developments in a wide range of clinical scenarios. We hope you enjoy it!

## Author contributions

The author confirms being the sole contributor of this work and has approved it for publication.

## Funding

Supported by grant P30-CA008748 (Craig Thompson PI).

## Conflict of interest

The author declares that the research was conducted in the absence of any commercial or financial relationships that could be construed as a potential conflict of interest.

## Publisher’s note

All claims expressed in this article are solely those of the authors and do not necessarily represent those of their affiliated organizations, or those of the publisher, the editors and the reviewers. Any product that may be evaluated in this article, or claim that may be made by its manufacturer, is not guaranteed or endorsed by the publisher.

